# An update on Uniform Resource Locator (URL) decay in MEDLINE abstracts and measures for its mitigation

**DOI:** 10.1186/1472-6947-8-23

**Published:** 2008-06-11

**Authors:** Erick Ducut, Fang Liu, Paul Fontelo

**Affiliations:** 1 Lister Hill National Center for Biomedical Communications, National Library of Medicine, National Institutes of Health, Bethesda MD, USA

## Abstract

**Background:**

For years, Uniform Resource Locator (URL) decay or "link rot" has been a growing concern in the field of biomedical sciences. This paper addresses this issue by examining the status of the URLs published in MEDLINE abstracts, establishing current availability and estimating URL decay in these records from 1994 to 2006. We also reviewed the information provided by the URL to determine if the context that the author cited in writing the paper is the same information presently available in the URL. Lastly, with all the documented recommended methods to preserve URL links, we determined which among them have gained acceptance among authors and publishers.

**Methods:**

MEDLINE records from 1994 to 2006 from the National Library of Medicine in Extensible Mark-up Language (XML) format were processed yielding 10,208 URL addresses. These were accessed once daily at random times for 30 days. Titles and abstracts were also searched for the presence of archival tools such as WebCite, Persistent URL (PURL) and Digital Object Identifier (DOI).

**Results:**

Results showed that the average URL length ranged from 13 to 425 characters with a mean length of 35 characters [Standard Deviation (SD) = 13.51; 95% confidence interval (CI) 13.25 to 13.77]. The most common top-level domains were ".org" and ".edu", each with 34%. About 81% of the URL pool was available 90% to 100% of the time, but only 78% of these contained the actual information mentioned in the MEDLINE record. "Dead" URLs constituted 16% of the total. Finally, a survey of archival tool usage showed that since its introduction in 1998, only 519 of all abstracts reviewed had incorporated DOI addresses in their MEDLINE abstracts.

**Conclusion:**

URL persistence parallels previous studies which showed approximately 81% general availability during the 1-month study period. As peer-reviewed literature remains to be the main source of information in biomedicine, we need to ensure the accuracy and preservation of these links.

## Background

Today, almost all major biomedical publications are accessible on the Internet. Researchers start out scanning the Internet for references and a quick scan of most print journal article will show references to Web sites. Many publications now have electronic versions on-line. Collaboration is often conducted on the Internet through Web 2.0 technologies such as mashups, Ajax and social networking applications. The biomedical community has accepted the Internet as the optimal medium where one can readily share formats not suitable for print media like high-resolution images, animations, videos, large databases and applications. Journals often provide "Web-only" additions to print publications. One could say that general acceptance, ease of use, and high bandwidth have all contributed to this electronic information "boom." For publishers, the Internet provides immediate worldwide dissemination not possible with traditional print media.

However, the fleeting nature of information on the Internet and rapid changes in Web technologies have both biomedical authors and publishers concerned [1]. The constantly changing environment of the Internet does not provide any guarantee of permanence. "The Internet Archive" estimates the average lifespan of a Web page as only about 77 days [2]. No data is currently available for biomedical Web sites.

Uniform Resource Locator (URL) decay or "link rot" has been a growing concern in the field of biomedical sciences [3]. In general, URL decay occurs for several reasons: servers may shutdown because of business failures; URL content may change or reconfigured by the Web site owners; and errors in URL citing may occur. This is especially true for long URLs that can easily get truncated [4]. Several studies have tried to document the extent of URL decay in scientific literature. A 2002 study reviewing Web site availability three years after it was initially accessed showed, "over two-thirds could not be found anymore or had moved with no forwarding URL" [5]. Wren in 2004 cited that only 78% of URLs published in MEDLINE abstracts were generally available at the time of accession [6]. Latest studies placed URL availability at about 70% with an annual decay rate of 5.4% [1]. A common conclusion in all reviews is a high URL decay rate for biomedical journals as well. Since the availability of a published URL is a function of time published, it is expected that inaccessibility of URL links will get worse unless measures are provided to alleviate the situation.

In order to reduce URL decay, several solutions were proposed. One is the use of Digital Object Identifiers (DOI) which are permanent, unique identifiers that can accompany any URL [7]. DOIs are given to an electronic document that, in contrast to a URL, are not dependent upon the electronic document's location. It is used to provide updated information, including the location of the object in the Internet, services about the object, or any other defined piece of data. Information about a digital object may change over time, but its DOI and hence its location will not change. DOIs can be used to identify e-texts, images, audio or video items, software and databases. CrossRef, an independent membership association founded and directed by publishers, operates a cross-publisher citation linking system that allows an end-user to click on a reference citation on one publisher's platform and link directly to the cited content on another publisher's platform [8]. Presently, this citation-linking network reportedly covers millions of articles and other content items from several hundred scholarly and professional publishers. This measure has still to gain wide acceptance with small publishers and individual authors because of its cost. Annual fees for publishers are dependent on the publisher's total publishing revenue [8].

Another method is the use of the Persistent Uniform Resource Locator or PURL, proposed by the Online Computer Library Center [9]. Like the DOI, PURL is a permanent, unique address. Any changes in the publishers content or organization of their material will only necessitate an update in the PURL server.

WebCite was developed to prevent "link rot" in scholarly journals [10]. It permanently archives and retrieves cited Internet references. Authors, readers, editors and publishers alike can use WebCite. It is offered free of charge to authors and readers while participating publishers are charged a membership fee similar to the CrossRef service. While the CrossRef model crosslinks between "traditional" published journal references, WebCite has the added advantage that it can archive "non-traditional" materials and Web sites. WebCite has more than 100 journals using its services since it started in October 2005. According to Gunther Eysenbach, the developer of WebCite, WebCite links are not commonly encountered in MEDLINE abstracts. Most recently, publishers have started submitting their papers to WebCite so that cited URLs can be archived immediately after publication. Links to WebCite usually appear on the publisher website, but not necessarily in the abstract or full text of the papers. URLs cited in the abstract may have been archived by WebCite, even though a WebCite link may not appear in the MEDLINE abstract (G. Eysenbach, personal communication, April 16, 2008).

Internet Archive and Google services are available to researchers encountering Internet references that are inactive. Internet Archive is a non-profit group that provides free access to a digital library of Internet sites [2]. Google provides cached Web pages [11]. These services, however, are far from ideal because they may only 'recover' a third to one-half of the total Web pages on the Internet [6,12]. In addition, the archived page may not be the actual Web page accessed and cited originally by the author, hence may not contain the actual information. Lastly, archived pages do not include large databases or applications.

This paper attempts to review the characteristics of the URLs published in MEDLINE abstracts; provide an update on the current status of the availability of these URLs; and estimate URL decay in MEDLINE abstracts over the years. The investigators also evaluated the information provided by the URL to determine if the information that the author cited in the abstract is the same information, i.e. the context it was used originally. Lastly, among the documented methods to preserve URL links, the investigators wanted to determine which ones have gained acceptance among authors and publishers. The paper does not attempt to evaluate or characterize decay of URL links in the actual full-text article.

## Methods

**Figure 1:**
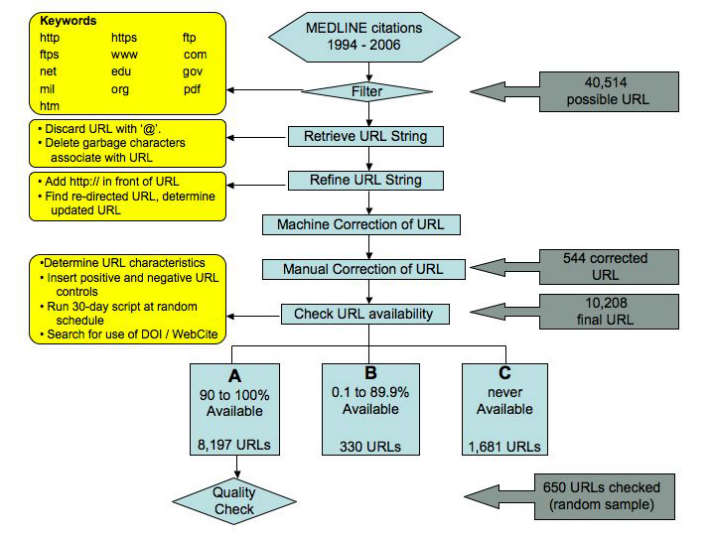
**Algorithm of URL decay study**. MEDLINE records from 1994 to 2006 were searched for the presence of a URL. 10,208 URLs were examined for the study. A random sample of 650 URLs were checked manually to determine accuracy of information in the Website.

The study was conducted from May to July 2007. MEDLINE records from the National Library of Medicine from 1994 to 2006 in Extensible Mark-up Language (XML) format were flagged for key terms that indicated the possible presence of a URL. Using PHP 5.2 scripts developed by the authors, URL addresses were extracted from the titles and abstracts of MEDLINE journals. The database included the PubMed ID (PMID), title of the paper, date of publication, name of journal and URL characteristics including length in number of characters, presence of international addresses and type of top-level domain. On the initial run, URL addresses were accessed using PHP scripts; addresses that returned a failed attempt were reviewed for formatting errors. PHP scripts were also used to determine the type of formatting error and correct the URL record if necessary – these included adding "http://" if it was not present, removing inappropriate spaces and erroneous characters, like back slashes. The investigators also did manual corrections when possible; these involved comparing the URL address in our database with the address in the MEDLINE title or abstract. Web sites that redirected to another page were noted and the updated URL addresses were used for the study [Figure 1].

As positive controls, 50 URLs known to be fully functional were added to the database. Ten false URL addresses were also added as negative controls. Using PHP 5.2 and Perl, URLs were accessed once daily, including weekends, at random schedules for 30 days. Attempts were recorded for each run and entered into the database. An attempt was considered a failure if the URL address did not return a Web page within 60 seconds, returned a "Page Not Found" message or if any other error message was returned. Twenty-five consecutive unsuccessful attempts were considered a failure, thus terminated the run. A repeat run was conducted 2 hours after the initial run if the previous run was terminated.

URLs were grouped into 3 categories. Grouping was based on a similar study by Wren in 2004 [6]. Group A consisted of URLs which were available 90 to 100% of the time during the 30-day study period. Group B were URLs with variable availability (greater than 0 to 89.9%). Group C URLs were not available at any time and were considered "dead" links.

Using PHP scripts, URLs from Group A were randomly selected by the computer algorithm (sample size = 650 URLs; alpha level = .05, p value = .50) and manually checked by the investigators for information quality, to ascertain that the URL contained the actual information as mentioned in the MEDLINE record.

Using the XML file, titles and abstracts were also searched for the presence of archival tools such as WebCite, PURL and Digital Object Identifier (DOI) using these names as keywords and their syntax format (e.g. http://www.webcitation.org/xxxxxxxx). We also examined the special field for DOI in the MEDLINE XML record.

## Results

**Figure 2:**
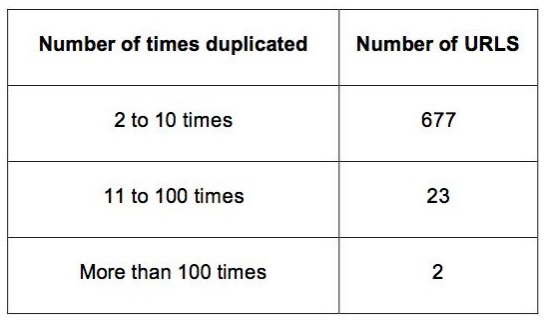
**Duplicated URLs and their frequency**. A total of 702 URLs were duplicated 2651 times. Most of the duplicate URLs were used twice.

A total of 40,514 MEDLINE citations were flagged for the possible presence of URLs. After removing abstracts that only contained e-mail addresses or had the key terms but did not have an actual URL address within the record, 10,208 URL addresses remained [see Additional file 1]. Seven hundred two URLs were determined to be duplicated and were cited in 2,651 different MEDLINE abstracts. A great majority of these URLs (677 out of 702) were cited less than 10 times [Figure 2].

All URL addresses were checked for errors during the initial run. A total of 2,245 URLs were not accessible during the initial run, thus necessitating manual review. Of this group, 544 URLs were found to have errors in formatting. Common errors in formatting include: extra spaces within the addresses, the use of backward slashes and the inclusion of erroneous characters. Manual correction of the URLs was done as needed by the investigators. A total of 163 URLs redirected to another page and the updated addresses were used in the study run.

URL length ranged from 13 to 425 characters with a mean length of 35 [Standard Deviation (SD) = 13.51; 95% confidence interval (CI) 13.25 to 13.77]. The most common top-level domains were ".org" and ".edu", each with 34% of the total URLs in the database. Other top-level domains were ".com" (18%), ".gov" (10%) and ".net" (3%). About 1% had a URL address that did not follow conventional domain formats. A total of 4,553 or 44.6% of the total URLs had top-level domains with addresses identified as being outside the United States.

Of the 30 daily runs, 2 had Internet connection failures from the investigators' end, which were repeated 2 hours after the initial run.

**Figure 3:**
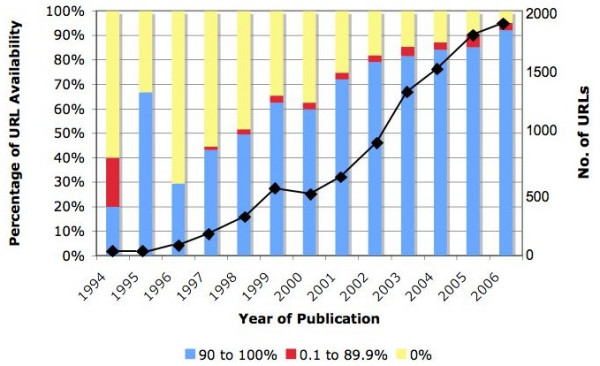
**URL Availability by Year of Publication**. 10, 208 URLs extracted from MEDLINE records from 1994 to 2006 were accessed once daily at random schedules for 30 days to determine link availability. The bar graph shows that URL availability seems to be time-dependent with those being published earlier showing a higher decay rate. The increasing number of URLs in MEDLINE abstracts by publication year (solid line) is also superimposed with the bar graph.

A total of 8,197 or 81% were available 90 to 100% of the time. The number of URLs with variable availability (greater than 0 to 89.9%) was 330 or 3%. Dead URLs constituted 1681 or 16% of the total. To facilitate comparison of results, this grouping was patterned after a similar study by Wren in 2004 [6]. Figure 3 shows the number of cited URL per publication year and illustrates that decay is higher for abstracts published at an earlier date. Removing duplicate URLs, URL availability (90 to 100% available) drops to 77.13%.

Seventy eight percent of the accessed URLs showed the actual information as mentioned in the MEDLINE record. Information in some of the URLs (20.9%) were not readily accessible because the URL either directed to the parent directory of the Web page or had a Web page in a non-English language hence information can not be easily ascertained or followed by the investigators. About 1.1% had no information available at all or had the wrong URL.

A review of the MEDLINE records from 1998 to 2006 for the presence of DOI in the PubMed citations showed that 519 abstracts had incorporated DOI addresses in their abstract using the special field for DOI provided in the MEDLINE record. No PURL or WebCite addresses were found in the MEDLINE titles or abstracts.

## Discussion

**Figure 4:**
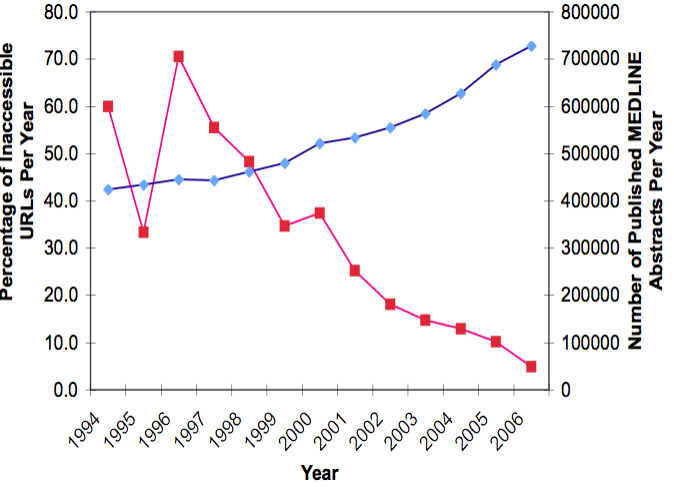
**Number of Published MEDLINE Abstracts per Year and the Percentage of Inaccessible URLs Accessed During the 30-day Period**. The graph shows the number of MEDLINE abstracts published per year (blue line) against the percentage of inaccessible URLs per year of publication (red line). The availability of a URL is dependent on time after publication. A higher decay rate is seen in earlier articles.

The number of URLs being published in MEDLINE records is growing each year but the constantly changing environment of the Internet does not provide any guarantee of permanence of these links [Figure 4]. Changing Web contents and Web addresses contribute significantly to URL decay. In addition, these Web practices do not guarantee that the information accessed by the author during the preparation of his paper is the same information that future readers or researchers will be able to access.

In this 30-day review, 81% of the total number of URL links in MEDLINE abstracts were accessible during the study. This rate closely approximates the rates of 78% by Wren in 2004 and 70% by Carnevale, et al. in 2007 [1,6]. These translate to about 20 to 30% loss of published biomedical links. Removing the duplicate URLs from the database would yield a lower URL availability (77.13%). The investigators, however, feel that there is value in reporting the availability of all the URLs including the duplicates because these duplicate URLs were cited by different articles and oftentimes in different time periods. Although the biomedical community has acknowledged URL decay as a problem, the rate of URL availability appears to have only slightly improved, if not remained the same, as this study has demonstrated. Perhaps improved infrastructure and Web technologies have contributed to the preservation and permanence in biomedical journals. However, because the number of published URLs is increasing tremendously each year, caution must be observed in analyzing this rate, as there is a bias towards more recently published URLs.

Since other researchers have reported that the availability of a URL is dependent on how long ago it was published with those being published early showing a higher decay rate, the questions that need to be raised are, "Are we going to expect to see more and more URLs become unavailable as years pass? [6]". Is permanence just a function of available infrastructure and technology or is it also a function of time? In short, can we be assured that the high availability of present URLs will remain the same 10 or 20 years from now?

The main function of a reference list is to provide support for statements put forward by the author of the paper. In our study, we sampled the URL list and compared the information found in the referenced Web site to that mentioned in the paper. Only 78% of the total sampled URL addresses showed the actual information that was accessed and used by the author. The rest directed to the parent directory of the Web site and required the readers to search for the information themselves within the Web site. For English speakers, this may pose as a challenge as some Web pages were published in non-English languages and did not offer any translations in English. In the biomedical field where accuracy and reproducibility are important, the reader must be confident that the article being linked or read is the exact article in the author's reference list. If the supporting material becomes unavailable or changes over time, the central function of a reference to provide factual support for the author's statements diminishes [13].

A URL indicates the location in the host server of materials posted on the Internet. Users can follow the link provided by the URL by clicking on it or typing the URL address directly into the browser. In our study, URL length ranged from as short as 13 characters to as long as 425 characters. URLs that are long are unwieldy and prone to errors in typing and formatting. Long URLs lead to truncations and errors. Any mistyped, omitted or added character in the URL during the preparation of the MEDLINE record will produce an error as was evident in our study. In contrast, DOI or WebCite links are usually less than 20 characters in length and are less likely to produce errors. Common errors that we found in the URL addresses in the MEDLINE abstracts include the presence of inappropriate spaces and back slashes and omission of necessary characters like slashes and colons that could have been added or omitted during the preparation of the MEDLINE record itself.

As part of our study, we searched for the presence of DOI, CrossRef, PURL and WebCite annotations in the MEDLINE records in our database. Though not exactly a metric of URL link preservation, the investigators believe that their use demonstrates an effort to preserve the links. We wanted to determine which among the proposed measures to mitigate URL decay have been accepted by the authors and publishers. A total of 519 MEDLINE citations incorporated DOI addresses in their records, found primarily in the special MEDLINE field allotted for DOIs. It is important to note, however, that these DOIs are used to reference the actual published article and not the cited URL links in the MEDLINE abstract. No PURL, WebCite or CrossRef addresses were found in the abstracts. Since WebCite and CrossRef citations are common nowadays and mention hundreds of affiliated publications, we believe that their use are more commonly found in the body of the full text rather than in the MEDLINE record (titles and abstracts only). This was confirmed thru an email correspondence with WebCite's founder, Gunther Eysenbach (G. Eysenbach, personal communication, April 16, 2008).

Abstracts, aside from the title, are the most easily accessible portion of a scientific article. Because of this, abstracts should accurately reflect the study both in specific data and overall message [14]. It is important to mention that some physicians may rely heavily on medical abstracts to guide their medical decisions. Because of the sheer number of published materials competing for their limited time available to stay current, practitioners may resort to reading the abstracts only and to rely on abstracts to stay informed [15-20]. Ward, et al. reported in their 2004 paper that "the abundance of published materials competing with the finite amount of time available to stay current with the literature may force many practitioners to resort to reading only the article abstract." In 2000, the Journal of General Internal Medicine published a study by Saint, et al. that surveyed the journal reading habits of physicians practicing internal medicine in the United States. In their study, respondents reported reading only the abstracts for 63% of the journal articles [21]. It is important therefore, that although medical abstracts are considered self-standing, authors and publishers must ensure the accuracy of their abstracts including the validity of their URL links.

**Figure 5:**
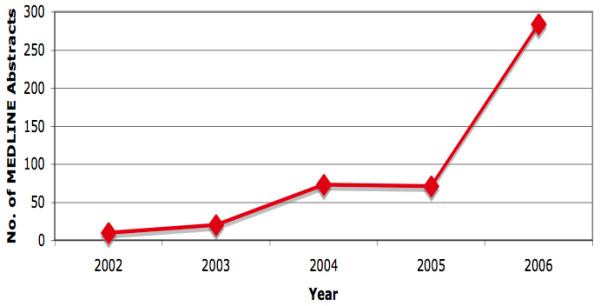
**Use of Direct Object Identifier (DOI) in MEDLINE abstracts by year of publication**. Only 519 MEDLINE abstracts with DOI addresses were found. No PURL or WebCite addresses were found.

Although the annual number of DOI addresses in MEDLINE abstracts have been increasing per year since its introduction in 1998, 519 is a very small percentage considering that more than 500,000 citations are added to MEDLINE each year. Acceptance of these archival tools is evidently increasing although not fast enough to cope with the growing numbers yearly [Figure 5].

Among the top-level domains, the combined number organizations (.org) and academic centers (.edu) addresses comprised 68% of the URL links. Any change therefore in Web practice, such as maintenance of Web servers and preservation of Web pages, in these two domains can significantly impact the rate of URL availability and decay. Though authors carry most of the responsibility in ensuring URL integrity, they are not always in the position to preserve it [5]. Academic centers and organizations are in a special position to make a significant impact and should therefore take on the responsibility and provide measures to ensure the availability of Web content.

We have provided an update of URL availability in MEDLINE abstracts only. Follow-up investigations should include an update of the availability of URLs, and the use of archival technology in actual journal references and in WebCite-enabled abstracts prepared by publishers. Previous studies have examined the content preservation of unavailable URLs in archiving sites such as, Google Cache and Internet Archive. Future studies can include another look at these Web services to determine its value in URL content preservation.

Publishers should probably discourage authors from citing URL links in their abstracts and full text, especially if they are long or contain too many characters. Publishers should instead suggest that authors who cite URLs in their papers should use Internet archival methods such as DOI and WebCite and recommend that MEDLINE use WebCite-enabled abstracts prepared by publishers.

## Conclusion

Our study has shown that although the rate of URL availability has remained the same through the years, it appears that neither significant effort nor progress has been made to mitigate URL decay rate. Perhaps, publishers could enforce the guidelines available for citing URL links in abstracts and full text, and recommend the use of archival tools should URL links be necessary. Our data raises more questions than the current study is able to address. Is the percentage of preservation or permanence of biomedical journals growing higher because of improved infrastructure or will it be a function of time or maybe both? Only time will tell. A re-look needs to be done to answer this question by repeating this study using the same set of URLs. This will determine the true decay rate of these URLs over time.

Publishers of biomedical journals have called on authors to do their share in preserving the scholastic integrity of their papers. As it is, the burden for editors and publishers continue to grow as the number of articles that are published each year are increasing tremendously. Though archival tools are readily available to the biomedical community, it seems that this practice has still yet to gain wide acceptance. As peer-reviewed literature remains to be the main source of knowledge in biomedicine, authors, academic centers, organizations, editors and publishers must heed the call to archive and share the task of preserving this digital literature. Efforts should be directed toward preserving and ensuring the permanence of biomedical journal archives. The tools to ensure permanence are available but the only way to ensure permanence is to exert the effort to achieve it.

## Competing interests

The authors declare that they have no competing interests.

## Authors' contributions

ED conceptualized the study design and drafted the manuscript. FL provided the programming framework to acquire and analyze the data. PF made significant contributions to the conception and design of the study and analysis of data. PF has also been involved in critically revising the manuscript for important intellectual content.

## Pre-publication history

The pre-publication history for this paper can be accessed here:



## Supplementary Material

Additional file 1 **Study Database**. This is the general database of URL addresses that were extracted from MEDLINE abstracts from 1994 to 2006. Click here for file
